# An option contract model for leasing containers in the shipping industry

**DOI:** 10.1057/s41278-020-00167-2

**Published:** 2020-09-28

**Authors:** Alejandra Gómez-Padilla, Rosa G. González-Ramírez, Fernando Alarcón, Stefan Voß

**Affiliations:** 1 Department of Industrial Engineering, Universidad de Guadalajara, Guadalajara, Mexico; 2 Faculty of Engineering and Applied Sciences, Universidad de los Andes Chile, Mons. Álvaro del Portillo 12455 Las Condes, Santiago, Chile; 3 Hapag Lloyd AG, Santiago, Chile and Hamburg, Germany; 4 Institute of Information Systems, University of Hamburg, Hamburg, Germany

**Keywords:** Container leasing, Option contracts, Cox–Ross–Rubinstein pricing model, Maritime shipping, Shipping line

## Abstract

We propose an option contract model for the leasing of containers. In an option contract, the shipping company commits to order a quantity of containers from the leasing company and has the right to modify its order at a later stage, according to its actual requirement. Under this scheme, the shipping company is allowed to request a smaller or larger number of containers than the agreed initial order. This is done by buying an option premium in advance from the container leasing company. We present numerical results for different scenarios based on information provided by experts in the industry. For the purposes of comparison, a nonoption contract scheme is also evaluated. According to our numerical results, an option contract is better under a scenario where demand is normally distributed with a large standard deviation. This scenario is commonly observed in practice due to the dynamism and volatility of the shipping industry. We conclude that, under an option contract scheme, the shipping company has more flexibility to adjust its demand for containers and to be requested from the leasing company, and this adjustment is compensated by an option price determined according to variations in demand.

## Introduction

Intermodal transport, powered by containers, has contributed significantly to the economic development of nations and to the enormous growth of world trade. Transportation of general cargo has undergone important changes due to the introduction of the container.^1^

As indicated by the United Nations Conference on Trade and Development (UNCTAD 2019), world maritime trade lost momentum in 2018, with volumes expanding more slowly than historical averages. This deceleration was due to trade tensions and tariff escalation, mainly between China and the USA. Furthermore, with the COVID-19 pandemic, the shipping industry is facing important challenges, with a significant decrease in world trade.

The shipping industry is a volatile and risky sector, characterized by freight rate instability, a high degree of financial commitment for investments in assets, strong competition among carriers (Notteboom et al. 2010), and remarkable concentration (at least in container shipping). In this regard, the UNCTAD (2019) also indicates that, currently, three alliances dominate the container shipping market and capacity deployed on the three major East–West trade routes: the 2M Alliance, the Ocean Alliance, and The Alliance. Furthermore, the top 10 container shipping lines, most of them members of these alliances, have a market share of 90%.

Container shipping companies must at all times have available a number of containers for their transport services; these can be owned or leased. Due to East–West trade imbalances, containers need to be continuously repositioned. Trade imbalances mainly occur in the Trans-Pacific and Europe–Asia trades for dry containers and in North–South flows for reefer containers. This results in very large volumes of containers to handle and the need to reposition containers from surplus to deficit areas. As a result, shipping companies suffer from significant repositioning costs that can amount to up to 27% of total container handling costs (Song et al. 2005; Stahlbock and Voß 2010). One of the important decisions of a container shipping company is how to manage its fleet of containers and trade imbalances, and in this context, whether to buy or lease containers (Haralambides 2017). According to Hoffmann et al. (2019), leasing companies accounted for 55% of container purchases in 2017, and although the fleet size of shipping companies increased by only 2.4%, their leased container stock increased by 6.7%. Several strategies have been employed by shipping companies to reduce operational costs and achieve economies of scale, including cooperative agreements to share investment risks (Caschili et al. 2014), efficient planning of container stock inventories and repositioning (Olivo et al. 2005; Vojdani et al. 2013; Dang et al. 2013; Moon and Hong 2016; Haralambides 2019), and decisions on repair and maintenance (Hoffmann et al. 2019), including leasing of containers. Although leasing containers may be more expensive operationally, leasing provides more flexibility to the shipping lines, enabling them to adjust to shortfalls and shortages of containers and to combine leasing and repositioning decisions, such that, for instance, containers can be picked up at high-demand locations. In addition, shipping lines may be limited in financial resources, such that fully owning the required number of containers is not possible, thus leasing of containers becomes more attractive (Lun et al. 2010). However, low manufacturing or leasing costs of containers could favor purchasing or leasing decisions rather than repositioning (Wong et al. 2015). Moreover, the introduction of foldable containers offers the possibility to reduce container handling costs (Stahlbock and Voß 2010; Moon and Hong 2016).

Contracts have been studied in literature from different points of view and for a very wide spectrum of applications. Option contracts originate from financial economics (Kawai 1983; Trigeorgis 1996). In supply chain management, such contracts allow buyers to hedge against price and demand fluctuations, providing more flexibility on the amount of items to purchase once demand information is available. In this regard, option contracts may benefit the whole supply chain and increase profits across all partners (Xu 2010).

Option contracts for supply chain coordination have been extensively studied (Tsay 1999; Barnes-Schuster et al. 2002; Fu et al. 2010; Gomez-Padilla and Mishina 2009). There are two types of options: unilateral and bilateral. Unilateral options consist of call options and put options. In a call option, the buyer is allowed to increase the order quantity, while in the put option, the buyer is allowed to decrease it. In bidirectional options, they are allowed to both increase or decrease the quantities, but the unit price is higher than in unilateral options.

In the present article, we propose a model with option contracts for leasing containers under a long-term contract. A shipping company, under this type of contract, commits to order a certain quantity of containers from the leasing company, and it has the right to modify its order according to actual requirements. In this way, the shipping company can request a larger or smaller number of containers than its initial order. For this, the company should buy an option premium in advance as part of the contract scheme. The problem lies in determining the value of this option premium that is payed to the leasing company as part of the flexible contract, so as to benefit both players.

We present a numerical experiment representing the real conditions of an operation of a shipping company, based on the experience of industry experts as well as of the authors of the paper. We consider four scenarios, representing the case of a shipping company operating in different regions, described in Sect. 4. Our results offer insights on the benefits expected to be gained by the shipping and leasing companies as well as by the entire supply chain.^2^

The remainder of this manuscript is organized as follows. Section 2 presents a literature review. Section 3 presents the problem description and the proposed model. Section 4 provides a numerical illustration of the model considering a set of scenarios to evaluate. The paper ends with some conclusions and recommendations for future research in Sect. 5.

## Literature review

We present a review of the state-of-the-art of contributions in literature concerning the design of contracts in maritime applications and, more specifically, the different contributions related to the leasing of containers.

Authors have investigated different types of contracts in the maritime industry. Some examples include the design of personal employment contracts as a conduit for individualization, to facilitate better employment relationships at ports (Evans and Hudson 1994). Fisher (2012) provides a study of contracts for ship construction and their impacts on ship design preparation, analyzing the most common contractual factors to be considered in the preparation of such designs (e.g., the contractual answer, i.e., that any question can be answered by the contract itself—main elements such as agreement, terms and conditions, etc.). García-Pita (2013) studies the limits between freight contracts, carriage goods by sea contracts, and volume contracts, highlighting that the difference between each contract relies on the corresponding terms and responsibilities defined.

Bu et al. (2010) analyze the shipping capacity option contract between carriers and forwarders. They incorporate in their analysis the costs of container repositioning. Their results show that the carrier’s expected profit is a concave function of reservation and execution prices, while for the forwarder, it is a concave function of option reservation quantity. The authors also note different effects of container repositioning costs on the optimal decisions of both carrier and forwarder. Hurley and Walker (2013) study the responsibilities, rights, and liabilities of owner, charterer, master, and third parties in a charter party, as dictated by the transportation documents and the charter party.

In terms of container leasing contracts, there are different arrangements, and one important element that is taken into account in negotiations is the lease return, as leasing companies need to avoid an excessive on-hire of containers in shortage areas and their return in surplus ones. Hence, on/off-hire fees as well as quotas allowing returns during a time period can also be included in contracts.

Leasing contracts can be classified into three groups (Rodrigue et al. 2013, adapted from Theofanis and Boile 2009): (1) master lease, (2) long-term lease, and (3) short-term lease. Master leases are also called full service leases or container pool management plans. Here, the leasing company assumes full management of the containers, including maintenance and repair as well as repositioning, following off-hire, and contract termination. Depending on location and equipment conditions, a master lease may consider on-hire and off-hire credits or debits. The leasing contract specifies a variable duration, which typically ranges between short and medium term. The contract may also specify a variable number of containers, setting a minimum and maximum number. As highlighted by Rodrigue et al. (2013), the leasing company acts as a logistics service provider in several ways under this contract. The different costs included in a master leasing contract are illustrated by Lun et al. (2010) and include container rental, depot lift-on/lift-off charges, on-hire/off-hire drayage, drop-off charge, and off-hire repair cost among others.

Long-term leases are also called dry leases and usually entail a 5–8-year contract. The leasing company does not have any management responsibility over the leased containers. The lease normally follows the purchase of new containers by the leasing company. As indicated by Rodrigue et al. (2013), recent trends involve a shift from master to long-term leases. Another characteristic of this type of lease is that containers are integrated into the fleet of owned containers of the carrier, which enables more effective container handling. Also, they have low business risks and lower rental turnover than master lease contracts. Another important feature is that, under long-term lease contracts, shipping lines have the option of direct interchange of containers with another carrier, something that provides flexibility in the repositioning of containers. In this case, the leasing company charges a fee that is established in the contract. Current trends consider a shift from master leases to long-term leases (Rodrigue et al. 2013).

Short-term leases are also called spot market leases, and their pricing is influenced by current market conditions. These contracts are commonly used when there is a temporary surge in demand. In general, leasing companies do not maintain a significant percentage of their containers on this type of contract due to the high level of risk (Theofanis and Boile 2009). In this case, the shipping company is also responsible for the container management and repositioning. Short-term contracts may be more expensive but offer more flexibility for the shipping company, and this may be a good option when demand fluctuates significantly.

Container leasing is a very important decision for shipping companies, and this can be addressed from two perspectives. One is related to the total volume of containers owned by the lessor and the share of leased containers among the world container fleet. However, few contributions on related decisions can be found in literature, mainly due to the difficulty of modeling the container leasing itself and the complex interaction between container leasing and other decisions (Dong and Song 2012).

The problem of liner shipping network design has been widely studied (Fagerholt 1999, 2004; Reinhardt and Pisinger 2012), and it has also been solved, integrated with the container repositioning problem (Shintani et al. 2007; Meng and Wang 2011). Hoffmann et al. (2019) propose a decision model for the repair and maintenance of damaged containers which determines whether a certain repair will pay off, and if not, what should be done next (secondary use, sell, or lease). A multicommodity dynamic network flow model for container repositioning on a liner shipping network was considered by Varshavets et al. (2013); in this study, the authors consider the case of short-term leasing options. Dong and Song (2012) address the container leasing term optimization problem. They propose a mathematical model and a simulation-based approach to determine the length of the leasing contracts at a tactical and short-term level, providing recommendations for designing appropriate lease terms in various situations. Zheng et al. (2016) address a liner shipping network design problem with empty container repositioning to measure the perceived container leasing prices at different ports.

The only article found in literature exploring an option contract leasing scheme is the one by Liu et al. (2013). These authors develop a two-echelon container shipping service contract with bidirectional options, in which the shipping line can adjust the order by both increasing or decreasing the number of containers. Their model includes capacity and fixed order constraints. They consider two profiles of shipping companies: an aggressive and a conservative carrier. The main difference with respect to previous works is that we consider only bidirectional options and not shipping capacity or limited orders of the carrier. Another difference concerns our modeling of prices. Liu et al. (2013) assume a fixed price per container, independently of the quantities finally leased by the shipping company. Here, we assume that the price to pay to the leasing company depends on the variation in the ordered quantity of containers and on the option premium. Demand is considered to be independent. Liu et al. (2013) assume a maximum capacity of the shipping line, which is not the case in our model. Finally, Liu et al. (2013) consider, in particular, a case in China, while our computational experiments consider different regions for the design of the scenarios tested as well as a sensitivity analysis.

## Container handling operations: background

Due to trade imbalances across different regions, containers must be repositioned to ensure availability at each node of the global transport chain. In this regard, a certain percentage of the containers transported by a vessel could correspond to containers for repositioning. This percentage can amount to up to 30% of the total TEUs (twenty foot equivalent units) transported.

Container shipping companies, as mentioned above, require a number of containers to support their transport services, and these could be a mix of leased and owned containers. At each port of call, carriers need to keep a stock of containers, and these need to be stored, maintained, and repaired. These services are generally provided in dedicated facilities, such as container depots or empty container parks. Container services may also be offered at port terminals (at least storage).

For the cases analyzed in this article, we consider a situation in which the shipping company uses a container depot (or several) for the handling of containers to support export and import operations at each port of call. Such container depots can be located either at the interport area or at the hinterland. When containers are repositioned (either in an export or import operation), the port terminal and the container depot need to coordinate the movement of a batch of containers from the terminal to the depot or vice versa (Pascual et al. 2017).

In the case of import containers, once a full container is unloaded from the vessel and stacked temporarily at the terminal yard, the consignee arranges either an appointment or the corresponding procedure to pick up the container, deconsolidate the cargo, and return the container to the container depot indicated by the carrier. Shipping lines usually define a maximum number of days allowed to return the container. If these days are exceeded, the shipping line imposes a penalty known as demurrage. At the depot, the container is received and inspected to verify that it is in good condition and does not require any repairs.

If the container is damaged, the container depot notifies the shipping line and requests authorization for its repair. The carrier may or may not authorize the repair; in the latter case, intending to transport the container to another port. This is because the costs of repair vary according to the place. For instance, according to the experience of one of the authors while working for the South American Steam Company [Compañía Sudamericana de Vapores (CSAV)], a major repair cost may be around US $300 per container in Europe, US $50 in China, and US $125 in South America. Values clearly may vary, but this can illustrate the magnitude of such differences. For this reason, shipping lines may not necessarily repair the damaged containers at the same depots where they are first received. A repair process may require approximately one week depending on the level of damage and repairs to be performed. If the container is in good condition, then it may require just a cleaning service or be directly stacked at the corresponding position of the yard. In either case, containers are usually classified according to their condition and quality into three to four categories. Containers without damage are classified as type A, which indicates that they are capable of transporting food cargo, perishables, and in general, fragile cargo. At the other end, containers that are seriously damaged are considered a total loss and discarded. A container for reposition may cost around US $500–700.

For an export operation, the procedure is the opposite. Once the shipper has booked the transport service with the shipping line, the latter indicates to the shipper the depot where he needs to pick up a container. The full container eventually reaches the port terminal where it is temporarily stacked until loaded aboard the corresponding vessel.

At the depot, the procedure to pick up an empty container is very simple. As soon as the truck arrives, it is directed to the yard, where a yard crane will place the corresponding empty container. In most depots, truck arrival times are random, but some depots have introduced a notification system (e.g., in Australian ports), whereby truck drivers notify the depot about their arrival a few hours earlier.

Shipping lines and container depots can establish different agreements to govern their container operations. The two main types of such agreements consider the following conditions:

- The depot charges a price per storage day that is typically linear. Prices range according to location. To illustrate the relative differences in prices per location, we can consider that prices range from US $1 to US $1.5 in South America, US $3 in Europe, and less than US $1 in China. In this case, storage costs are linear.
- The depot offers a certain period as free storage or a free number of containers (free pool), and beyond those limits, additional days or additional containers are charged at about US $3–4 per day.

Hence, dispatching container policies vary. For instance, if the shipping line has a free-storage period, then it would normally request a first-in first-out (FIFO) policy for the dispatching of containers to external parties, as this would minimize storage costs. However, this may require some housekeeping operations at the depot, as containers with higher dwell times—that ought to be dispatched first—are most probably stacked below those with lower dwell times.

## Options contract for leasing containers: background and model proposal

We consider the problem faced by a shipping company that has to define the characteristics and terms of an options contract with a leasing company for leasing containers. In our case, the leasing company allows the carrier to modify the ordered number of containers by paying an option premium.

**Fig. 1:**
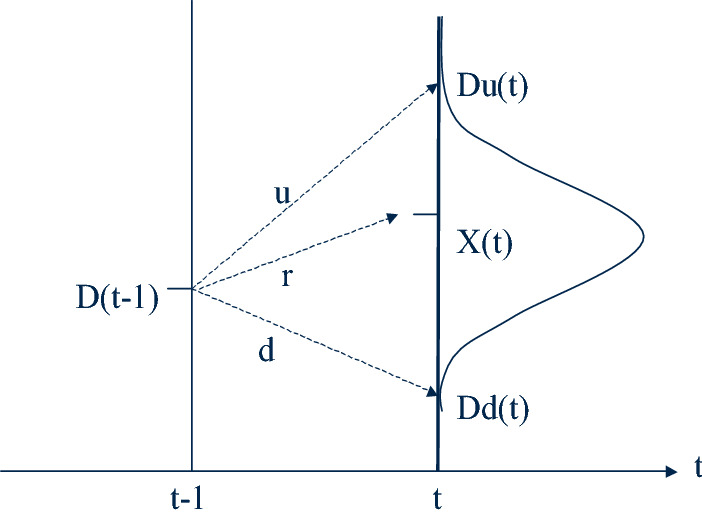
Expected demand at period *t*

At each period of time *t*, demand for containers by a shipping company is an expected value *X*(*t*). Demand data are important to calculate the option premium; this is calculated considering four parameters: (1) actual demand in the previous period of time: *D*(*t − *1); (2) average growth rate of demand: *r*; (3) upper limit of demand growth: *u*; and (4) lower limit of demand growth: *d*, where *d* ≤ *r* ≤ *u* and *d* < *u*. The average growth rate and the limits of demand growth rate should be negotiated by the two companies according to their experience, their statistics, and/or prospective analyses. The higher expected demand at time *t*, *Du*(*t*), and the lower expected demand at time *t*, *Dd*(*t*), are calculated with these parameters. The demand at time *t* depends on the demand of the previous period (*t* − 1), as shown in Fig. 1.

*Q*(*t*) is defined as the number of ordered containers by the carrier to the leasing company at time *t*. The inventory holds of the shipping company at time *t*, IS(*t*), will serve to satisfy demand of the shipping company. BS(*t*) is the stockout or unsatisfied demand by the shipping company at time *t*. *S*(*t*) represents the number of containers used by the company at time *t*. The company has a profit *p*(*t*) per container used at time *t*. In the model, we consider a cost associated with making the container available to use; this cost may, for instance, regard the repairing of the container in case the latter is needed at time *t* and is: *cS*(*t*). The inventory holding cost of the shipping company at time *t* is hS(*t*). The opportunity cost at time *t* is βS(*t*). TS(*t*) is the amount to be paid or transferred from the shipping company to the leasing company at time *t*. This amount is named “transfer” herein.

The leasing company bears the cost of making the container available (maintenance): cL(*t*). The shipping company and the leasing company have fixed costs linked to the execution of the contract represented by FCS and FCL, respectively. πS(*t*) is the profit of the shipping company at time *t*. The profit of the leasing company at time *t* is πL(*t*). The profit of both is *∏*(*t*), and it is just the sum of the two profits; this is the profit of the container supply chain formed by the two companies.

The profit for the shipping company is calculated from its revenue, minus the costs (making the container available, holding inventory, fixed costs, and transfer to the leasing company), as expressed in Eq. (1):

1 $$\pi S\left(t\right)=p\left(t\right)S\left(t\right)-\mathrm{cS}\left(t\right)Q\left(t\right)-\mathrm{hS}\left(t\right)\mathrm{IS}\left(t\right)-\mathrm{FCS}-\mathrm{TS}(t).$$

The benefit for the leasing company is calculated from the revenue received from the transfer from the shipping company minus its costs (cost of making the container available to lease and fixed costs). This is expressed in Eq. (2) as:

2 $$\pi L\left(t\right)=\mathrm{TS}\left(t\right)-\mathrm{cL}\left(t\right)Q\left(t\right)-\mathrm{FCL}.$$

The profit for the supply chain as a single unit is independent of the transfer from the shipping company to the leasing company, but the profit of the shipping company and of the leasing company strongly depend on this transfer, and the transfer depends on the contract. With an options contract, there is one price per unit leased, wS(*t*), and an option premium, OPS(*t*), to be paid according to the variation in demand provision from the initial to the final order.

The profit from leasing a container must be larger than the prices for the shipping company, $p\left( t \right) > {\text{wS}}\left( t \right) + {\text{cS}}\left( t \right),$ to obtain benefits.

### Option contract

In view of demand uncertainty, carrier and container lessor will agree to accept modifications in the ordered number of containers when they have a clearer idea about demand.

Call and put options therefore exist. When demand is higher than the initial estimate, the shipping company exercises a call option, since it will order more than initially planned. When demand is smaller than the initial estimate, a put option will be exercised by the shipping company, since the order will be smaller. To modify the ordered quantity, the shipping company will have to pay an option premium OPS(*t*) to the leasing company.

The option premium for a call option is

3 $${\text{OPS}}^{{{\text{C}}}} (t) = C_{{\text{R}}} \frac{1}{r}\left[ {\frac{r - d}{{u - d}}{\text{Max}}\left( {Du(t) - X(t),\;0} \right) + \frac{u - r}{{u - d}}{\text{Max}}\left( {Dd(t) - X(t),\;0} \right)} \right].$$

The option premium for a put option is

4 $${\text{OPS}}^{{{\text{P}}}} (t) = C_{{\text{R}}} \frac{1}{r}\left[ {\frac{r - d}{{u - d}}{\text{Max}}\left( {X(t) - Du(t),\;0} \right) + \frac{u - r}{{u - d}}{\text{Max}}\left( {X(t) - Dd(t),\;0} \right)} \right].$$

Equations (3) and (4) come from the Cox–Ross–Rubinstein pricing model (Cox et al. 1979). With this, it is possible to establish a sum that considers not only the difference between the expected demand and the actual demand but also three parameters that should be previously discussed and that will refine the result. This way, the option premium is proportional to the difference between these parameters and the actual demand. The conversion rate *C*_R_ gives the economic dimension to the result. The rest of the parameters (*d*, *u*, *r*) have to do with the demand; the conversion rate represents how much the demand variation will be magnified or diminished and is expressed in a sum to be paid for having the right of modifying the number of containers. The transfer from the shipping company to the leasing company with an option contract is given by the leasing price and the option premium:

5 $${\text{TS}}\left( t \right) = {\text{wS}}\left( t \right)Q\left( t \right) + {\text{OPS}}\left( t \right).$$

The price offered by the leasing company ought to be higher than its cost [wS(*t*) > cL(*t*)].

The profit for the chain is expressed as follows:

6 $$\prod \left( t \right) = p\left( t \right)S\left( t \right){-}{\text{cS}}\left( t \right)Q\left( t \right){-}{\text{hS}}\left( t \right){\text{IS}}\left( t \right){-}{\text{FCS}}{-}{\text{cL}}\left( t \right)Q\left( t \right){-}{\text{FCL}}.$$

## Numerical illustration

In this section, we present numerical results to illustrate the application of the proposed methodology. We divide the section into two parts. Section 5.1 presents four case studies that resemble real-life situations faced by a shipping company involved in operations in three regions. Section 5.2 presents a sensitivity analysis.

### Experiments resembling real-life situations

Consider a shipping company which leases containers from a leasing company. Based on the experience of one of the authors of this paper while working for the shipping line CSAV, we consider the following conditions to illustrate the application of the proposed methodology. Four scenarios are analyzed. The price at which the shipping company rents containers is *p*(*t*) per container at time *t* and is different in each case or scenario as described below. cS(*t*) is determined considering the average cost of repairs and that about 20% of containers are repaired. hS(*t*) is calculated by considering a linear holding cost per day—without free pool—and an average of 15 days per month. The price per unit leased, wS(*t*), is $24 per month, since the leasing cost is $0.80 per day, and we decided to standardize 30 days per month; this is a constant value in the four scenarios studied. We assume a null value cL(*t*) as well as the fixed costs FCS and FCL, because such costs are considered as part of the variable costs. The four scenarios were simulated using visual basic 7. We ran 10 simulations for both cases: with an option contract and without it. In the case of option contract, a conversion rate *C*_R_ = 1 was considered. Computational times were very slow, taking around 5 s each simulation. All the numerical experiments where performed using an AMD FX Eight Core Processor 4.01 GHz, 16 G RAM, Windows 7 ultimate 64 bit.

We assume that the leasing company has no capacity constraints in providing containers. In practice, this assumption may not be realistic. However, this may be overcome with the strategies employed by the leasing company regarding the special prices offered for recovering/handling containers in specific ports. This is not included in our analysis. Another limitation is that, for the nonoption contract scenario, we do not include charges or penalties for ordering less or more containers.

The values considered for each of the four scenarios are presented in Table 1. The values are based on the experience of one of the authors of the present paper while working for CSAV. We consider three regions for the analysis: South America, Europe and North America, and Asia. Europe and North America are taken together since they have similar values. One scenario is determined for each region. Another scenario was defined considering the average values.

**Table 1:** Data for simulated cases based on three regions analyzed

Parameter	Average	South America	Europe and North America	Asia
*p*(*t*)	300	50	500	300
cS(*t*)	20	25	60	10
hS(*t*)	15	19	45	12
wS(*t*)	24	24	24	24

The results presented show an average of 10 simulations for 12 months.

In addition, five demand patterns were considered: (1) uniform [0, 500], (2) slowing demand starting with uniform [0, 1000], (3) slowing demand starting with uniform [0, 500], (4) normal distribution with mean *μ* = 500 and standard deviation *σ* = 200, and (5) slowing demand starting with normal distribution with mean *μ* = 500 and standard deviation *σ* = 50.

**Fig. 2:**
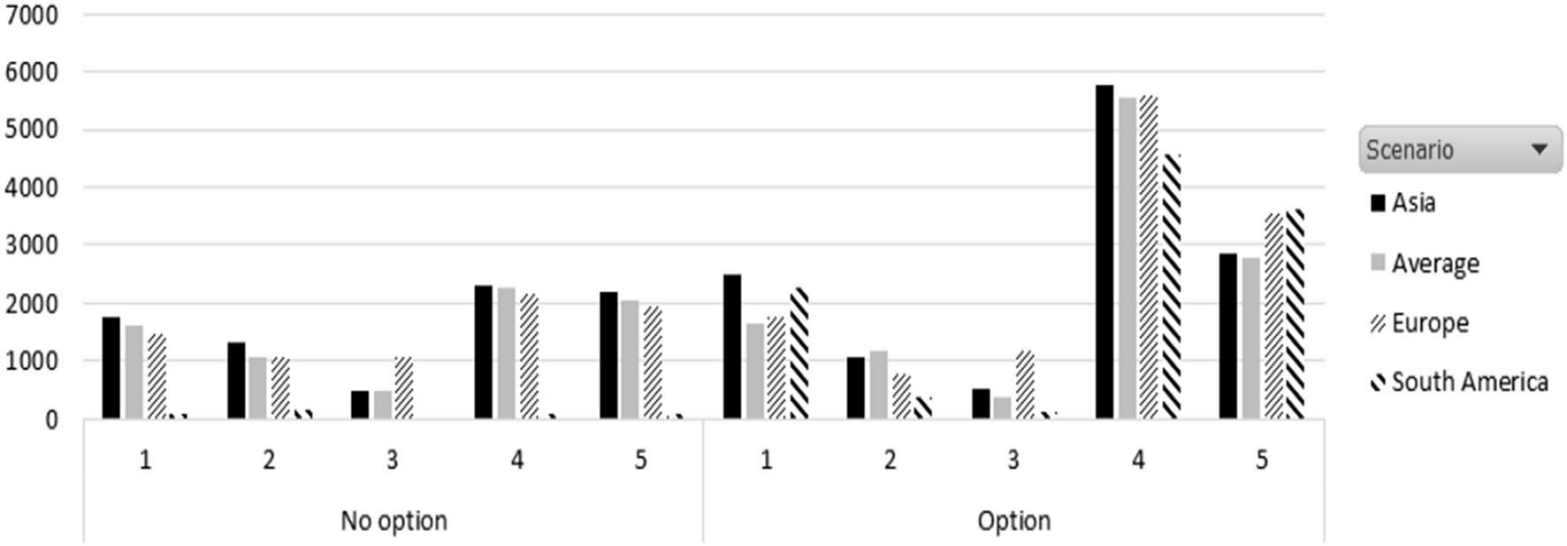
Leased units per demand pattern and scenario under nonoption and option contract

As shown in Fig. 2, the units leased are higher using an option contract in all demand patterns. This is easy to understand since, in an option contract, the final order can be modified once a more accurate demand expectation is available. An option contract is better especially under a demand pattern of normal distribution with high standard deviation, corresponding to pattern (4) in our case.

**Fig. 3:**
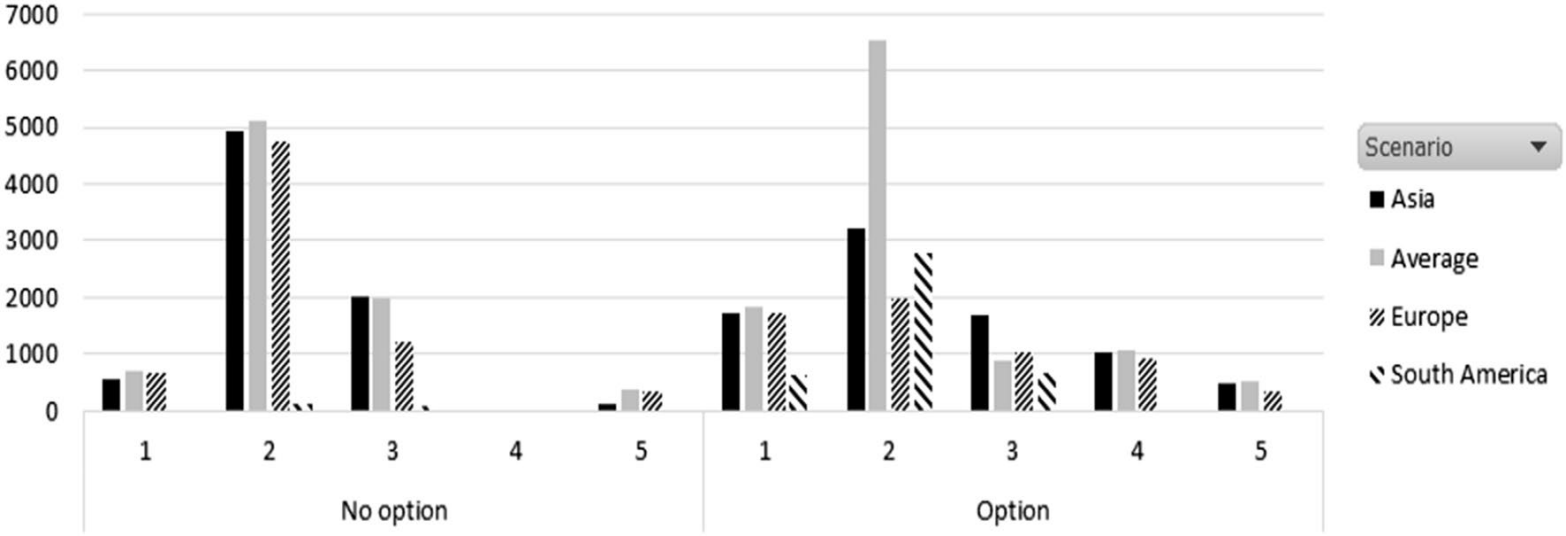
Stock per demand pattern and scenario under nonoption and option contract

Figure 3 shows that, with an option contract, the inventory of the shipping company increases; in two of the scenarios, the stock is higher than with a nonoption contract. Under decreasing demand starting with uniform, the inventory hold was higher in the average and South America scenarios.

As a result of holding more stock, under an option contract, lost sales are lower. This is explained because, with an option contract, more containers are leased. Figure 3 shows the net profit for the shipping company. We can appreciate that, in terms of net profit, an option contract is interesting only in the case of normally distributed demand. The European and North America scenario is the one with the higher profit, while South America has the lower profit. This is because the price *p*(*t*) is 10 times higher in Europe and North America than in South America, according to our scenarios.

**Fig. 4:**
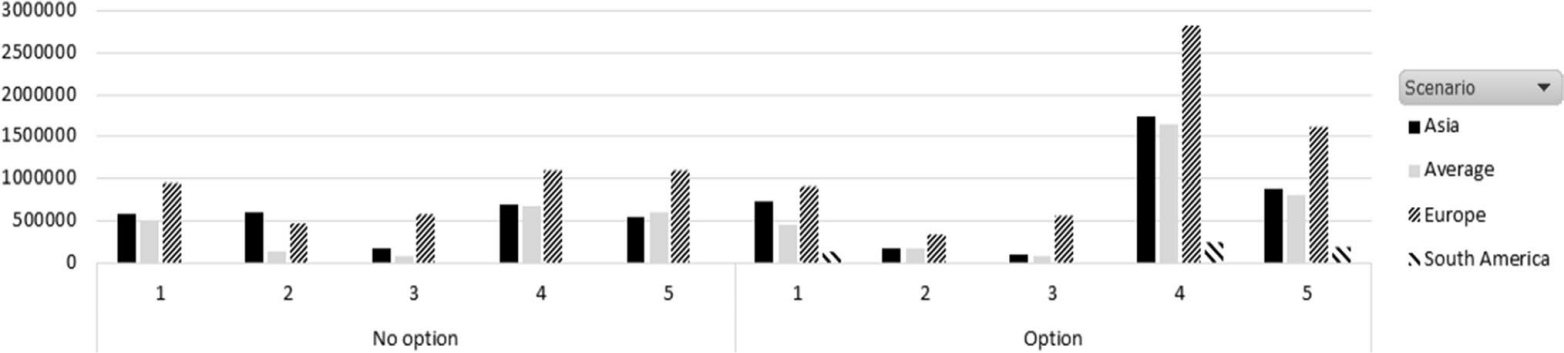
Net profit for the shipping company per demand pattern and scenario under nonoption and option contract

We can see from Fig. 4 that South America will not improve its profit with an option contract. This is explained as the net profit in this case decreases in a nonoption contract scenario. This is consistent with the results shown in Figs. 2 and 3, in which the South America case has the lower profit due to a lower price than in the rest of the cases.

### Sensitivity analysis

In this section, we present a sensitivity analysis to complement the numerical illustration provided above. For this, we vary the values of the parameters *p*(*t*), cS(*t*), and hS(*t*). In total, 16 combinations were considered, as presented in Table 2. The price per unit leased wS(*t*) = 1 in all cases. The other parameters are expressed in terms of wS(*t*); For example, in case 1, wS(*t*) = 1, the price of rent is twice that of wS(*t*), and the cost of repair and the holding cost amount to half wS(*t*). The results show an average of 10 simulations for 12 months. In Table 1, the cases reflect situations of moderate to very high difference between the leasing price and the price of rent, and also consider different repairing and holding costs. Case 2 represents a situation where the shipping company faces low costs, but it also has a low margin. Case 8 is a situation with moderate margin and high costs. Case 9 is a very advantageous situation of high margin and low costs, and case 15 is a fair situation of very high margin but also very high costs.

**Table 2:** Sensitivity analysis data for 16 cases

Case	wS(*t*)	*p*(*t*)	cS(*t*)	hS(*t*)
1	1	2	0.5	0.5
2	1	2	1	1
3	1	2	2	1.5
4	1	2	3	2
5	1	5	0.5	0.5
6	1	5	1	1
7	1	5	2	1.5
8	1	5	3	2
9	1	10	0.5	0.5
10	1	10	1	1
11	1	10	2	1.5
12	1	10	3	2
13	1	20	0.5	0.5
14	1	20	1	1
15	1	20	2	1.5
16	1	20	3	2

**Fig. 5:**
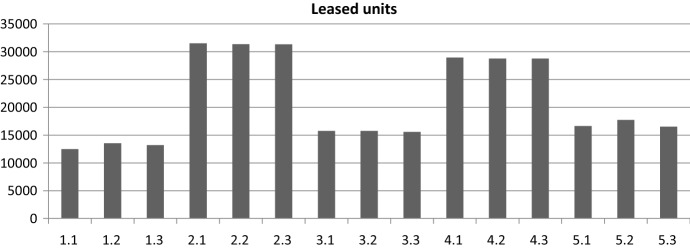
Leased units per demand pattern

In addition, five demand patterns were considered: (1) uniform [0, 500], (2) slowing demand starting with uniform [0, 1000], (3) slowing demand starting with uniform [0, 500], (4) normal distribution with mean *μ* = 500 and standard deviation *σ* = 200, and (5) slowing demand starting with normal distribution with mean *μ* = 500 and standard deviation *σ* = 50. Three conversion rates were considered: *C*_R_ = 0.5, 1, and 2. The 16 cases were simulated under the five demand patterns and the three conversion rates. As shown in Fig. 5, the units leased are higher for demand patterns (2) and (4). An option contract is better, especially under a demand pattern of uniform distribution with slowing demand and normal distribution with high standard deviation, corresponding to patterns (2) and (4) in our case.

**Fig. 6:**
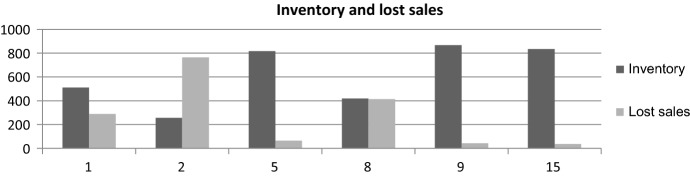
Inventory and lost sales for demand pattern 2 and conversion rate 1 (2.2)

Figure 6 shows that, with an option contract, the shipping company has high inventory in most of the cases. When its margin is low, the company may have lost sales, since it holds a low inventory. For Fig. 3, the case of demand pattern 2 and conversion rate *C*_R_ = 1 was considered, since all demand patterns have the same tendency.

**Fig. 7:**
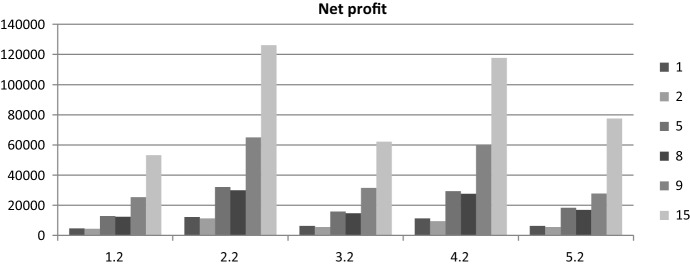
Net profit for shipping company per demand pattern for selected cases

As a result of holding more stock, under an option contract, lost sales are lower. This is explained because, with an option contract, more containers are leased. Figure 7 shows the net profit of the shipping company. We can appreciate that, in terms of net profit, an option contract is more interesting for demand patterns 2 and 4, i.e., in cases of very unstable demand. The cases with high margin and high costs are the ones with the higher profit.

## Managerial insights

According to our experience and that of experts in the area, leasing companies lease their containers on an annual or longer-term basis. In these contracts, leasing companies commit to make a number of containers available, while shipping companies engage to lease this number of containers during the length of the contract. If the shipping company finally orders fewer containers, a penalty is charged. Contrarily, the containers, if available, will be charged a higher price. Sometimes, leasing companies, knowing the itineraries of the shipping companies, may offer special prices for recovering and/or handling containers from and to specific locations. We propose a contract in which shipping companies determine a first approximate container demand per month and then place their final order. The modifications of this final order are settled by paying an option premium. In addition to the fluctuations in the demand for containers, shipping companies also need to renew their fleet of containers when a container’s useful economic life gets to its end. If this renewal process is planned in advance, more beneficial contract terms can be negotiated between the shipping line and the leasing company. We can mention CSAV as an example of a shipping line which, when it was operating, implemented option contracts as part of its strategy of leasing containers.

## Conclusions and further research

We propose a bidirectional option contracts model for the issue of leasing containers. Two stakeholders are involved: a shipping company and a leasing company. The issue considers the decision regarding the quantity of containers to be leased. Under an option contracts model, the shipping company is allowed to eventually request a bigger or smaller number of containers than its initial order, buying in advance an option premium.

Numerical results considering different scenarios that represent real-life situations faced by a shipping company during a planning horizon of 12 months allowed us to compare an option contracts approach with the baseline scenario where nonoption contracts are used. From the results, we can conclude that the use of option contracts is an interesting strategy in cases where demand approximates a normal distribution. In cases of uniform or decreasing demand, option contracts do not significantly increase profits. In practice, the assumption of a uniform distribution is less likely to occur; a reason why an option contract approach may be suitable. Furthermore, in the case of demand following the normal distribution, it was possible to obtain higher sold units as well as lower lost sales. Our results indicate that an option contract should be considered when demand is normally distributed with a high standard deviation. These results show the advantage of an option contract under a demand of high variability, which is the case of the global shipping industry. By using this strategy, leasing companies are able to provide a more flexible service, and in exchange, they will receive compensation (option premium) for the changes and the adjustments in the final order.

These results show the advantages of option contracts. In contrast with the results of Liu et al. (2013), our experiments did not relate call options with shipping capacity and the put option with the lower limit of orders. Hence, we do not limit the orders of the carrier: the objective of our research is to determine the demand patterns under which this type of options would represent an advantage.

As further research, additional numerical testing should be carried out based on other practical case studies. Different values for the conversion rate could be evaluated as well as other constraints such as the capacity of the leasing company (its number of containers to lease). Other charges that may be commonly employed in practice can also be considered (e.g., fee for returning or picking up containers at specific locations). Another extension could be to model the problem based on a bilevel approach for the contract design between the shipping and the leasing companies.

## Footnotes

1 The story of the container dates back to April 26, 1956, when the tanker ship Ideal-X sailed from Newark to Houston with 58 aluminum truck bodies aboard (Levinson 2016).

2 Preliminary results were presented at the International Association of Maritime Economists (IAME) conference, held in Hamburg in 2016 (Gomez-Padilla et al. 2016). Experiments and scenario configurations are extended in the present paper to provide more complete tests.
